# Linguistic analysis of empathy in medical school admission essays

**DOI:** 10.5116/ijme.5f2d.0359

**Published:** 2020-09-18

**Authors:** Mary Yaden, David Yaden, Anneke Buffone, Johannes Eichstaedt, Patrick Crutchley, Laura Smith, Jonathan Cass, Clara Callahan, Susan Rosenthal, Lyle Ungar, Andrew Schwartz, Mohammadreza Hojat

**Affiliations:** 1 Department of Psychiatry at the University of Pennsylvania, Philadelphia, PA, USA; 2 Department of Psychiatry and Behavioral Sciences at Johns Hopkins Medicine, Baltimore, MD, USA; 3 Department of Psychology at the University of Pennsylvania, Philadelphia, PA, USA; 4 Sidney Kimmel Medical College at Thomas Jefferson University, Philadelphia, PA, USA; 5 Department of Computer and Information Science at the University of Pennsylvania, Philadelphia, PA, USA; 6 Department of Computer Science at Stony Brook University, Stony Brook, New York, USA

**Keywords:** Medical education, admission, empathy, patient-centered care, linguistic analysis

## Abstract

**Objectives:**

This study aimed to determine whether words used in medical school admissions essays can predict physician empathy.

**Methods:**

A computational form of linguistic analysis was used for the content analysis of medical school admissions essays. Words in medical school admissions essays were computationally grouped into 20 'topics' which were then correlated with scores on the Jefferson Scale of Empathy. The study sample included 1,805 matriculants (between 2008-2015) at a single medical college in the North East of the United States who wrote an admissions essay and completed the Jefferson Scale of Empathy at matriculation.

**Results:**

After correcting for multiple comparisons and controlling for gender, the Jefferson Scale of Empathy scores significantly correlated with a linguistic topic (*r* = .074, *p*< .05). This topic was comprised of specific words used in essays such as "understanding," "compassion," "empathy," "feeling," and "trust." These words are related to themes emphasized in both theoretical writing and empirical studies on physician empathy.

**Conclusions:**

This study demonstrates that physician empathy can be predicted from medical school admission essays. The implications of this methodological capability, i.e. to quantitatively associate linguistic features or words with psychometric outcomes, bears on the future of medical education research and admissions. In particular, these findings suggest that those responsible for medical school admissions could identify more empathetic applicants based on the language of their application essays.

## Introduction

Initiatives to improve interpersonal aspects of patient care often forefront the empathy of medical providers.^1^ Empathy has been variously defined, with a rich theoretical and empirical literature.^2^^-^^5^ In patient care contexts, physician empathy has been defined as a predominantly cognitive attribute to understand patient experiences, combined with a capacity to communicate this understanding to patients, and an intention to help.^6^^-^^7^ Physician empathy measurably affects patient outcomes. Decades of theoretical and qualitative work in physician empathy preceded the development of quantitative measures of physician empathy,^8^^-^^10^ such as the Jefferson Scale of Empathy (JSE).^7^^,^^11^  Illustrative examples of empathy impacting patient outcomes include diabetic patients under the care of more empathic physicians have lower rates of metabolic complications.^12^ Diabetic patients who were treated by more empathic physicians show better control of their disease indicated by the results of laboratory tests of glycemic control and cholesterol.^12^ More empathic physician communication is also associated with improved patient satisfaction and patient compliance.^13^  Further, medical students with higher empathy do better during their clinical clerkships^14^ and are rated as more competent in the patient encounters.^15^ In sum, there is good evidence that physician empathy is linked to better patient outcomes and patient satisfaction. Research indicates that both patients and clinicians can benefit from empathic engagement.^6^

Teaching empathy has been included in the curriculum of several medical schools. Over the past few decades, educational programs have been initiated with varying degrees of success.^16^^-^^21^ Medical humanities programs have also been implemented with the intention of increasing empathy.^22^ Despite these initiatives, a significant drop in empathy occurs during the third year of medical school.^23^

Some medical educators have suggested that empathy should be included as a selection criterion for medical school applicants.^24^^,^^25^ Similar proposals have been made to adjust selection criteria for applicants with interest in primary care^26^ or to favor those with some background in the arts or humanities.^27^ However, self-reported measures of empathy can be influenced by social desirability response bias. An unobtrusive measure of physician empathy would be ideal, but developing unobtrusive measures presents a methodological challenge. A knowledge gap exists within medical education for measuring traits such as empathy using methods other than self-report.

Computational linguistic analysis, a quantitative method of corpus analysis, has been used in recent years to predict health issues such as heart disease mortality at the county level using language from posts on Twitter.^28^ A number of demographic and personality characteristics have also been explored using this technique with language from Facebook posts.^29^ In general, in cases where a link exists between language data and an outcome variable of interest in a given population, then this linguistic analysis method can identify the words that most correlate with scores on a given outcome measure.

In this study, we aimed to identify the words used in medical school admissions essays that are associated with self-reported physician empathy. The association of language use with physician empathy fills a knowledge gap by determining whether high empathy applicants can be detected through words used in medical school application essays. Our objective was to provide particular words from admissions essays that are most predictive of physician empathy.

## Methods

### Procedures

We used data from The Jefferson Longitudinal Study of Medical Education, an on-going study that surveys medical students on a yearly basis across a number of topics, including physician empathy.^30^ We also requested and received permission from the Association of American Medical Colleges (AAMC) to use medical school application essays written by the study participants. The texts of the essays were then merged with the Jefferson Longitudinal Study (Jefferson scale of empathy scores and demographics). This study was approved by the Thomas Jefferson University institutional review board (IRB).

### Study participants

Research participants included *N*=1,805 matriculants to Sidney Kimmel Medical College at Thomas Jefferson University between 2008-2015 who completed a survey, including a measure of physician empathy, at the beginning of medical school. This sample represents 85% of all matriculants (2,118) during that time period.

The study sample comprised 893 (49.5%) men and 912 (50.5%) women, with a mean age of 23.5 years. The gender composition and age of the study sample were similar to the total matriculants in the study period. Due to the reduced reliability of entries with lower word counts, participants must have written at least 500 words in their essays to be included in the sample. The 500-word cut-off also removed applicants from the sample for whom a full personal statement was not required.

### Instruments

Jefferson Scale of Empathy: We used the Jefferson Scale of Empathy (JSE), a 20-item, validated instrument specifically developed to measure empathy in the context of patient care in medical and other health professions students and practitioners. We used the 'S-version' of the JSE, which was developed for administration to medical students. Evidence in support of the JSE's validity and reliability^6^^,^^11^^,^^14^ has been reported. The possible score range is 20 to 140; a higher score on this scale indicates a greater orientation toward empathic engagement in patient care. The typical Cronbach's alpha for this instrument, which has been reported in many studies, is around .75.^6^ A sample item on this scale is: "It is difficult for a physician to view things from patients' perspectives." The JSE was completed by all of the medical students in this sample at matriculation.

### Data analysis

We used the process of Differential Language Analysis (DLA)^29^ to automatically identify clusters of words associated with a given outcome. DLA proceeds in two steps: (1) linguistic feature extraction – quantifying how often groups of words were mentioned and (2) correlation analysis – finding the association of linguistic features with given outcomes. The analysis was carried out within the computerized analysis program, Differential Language Analysis ToolKit,^32^ and the specific methods for each step that follow. DLA has been used previously in a number of studies to predict population health issues as well as explore linguistic correlations of personality and gender.^28^

For linguistic feature extraction, we first broke the admissions essays into words using DLATK's tokenizer, which separates sentences into words by spaces or other white space and punctuation.^31^ Based on a tokenized version of the entire corpus of essays, we then grouped words into related clusters, known as "topics", using two well-established topic modeling approaches: Latent Dirichlet Allocation (LDA)^32^ and Non negative Matrix Factorization (NMF).^33^ These statistical techniques find words that often appear in essays with the similar linguistic context. This approach leverages the individual advantages of LDA and NMF, allowing LDA to produce coherent topics when they are larger in number^34^ and for NMF to reduce dimensions while maintaining variance of count data effectively.^35^ In other words, while LDA is often run alone to produce hundreds of topics, our relatively small sample size limited our statistical power and required a lower number of linguistic variables. More broadly, these techniques reduce a very large number of words to a limited set of language variables called topics, which are comprised of words that share semantic similarities.^32^

Based on power analyses for effect sizes of *r* > 0.05, we calculated that approximately 20 topics as variables were sufficient while also correcting for false discovery rate in our significance tests.  At the end of this process, for each essay, we then have a usage score for the 20 topics which can be interpreted as the relative amount the topic words were mentioned within the essay.

For correlation analysis, the usage scores for the 20 topics were then treated as independent variables and were then associated with scores on the JSE, which was the dependent variable, using multivariate linear regression. Specifically, ordinary least squares linear regression was used with input variables standardized and with gender included as a covariate since it has been shown to be a significant factor in empathy in previous research.^36^ The correlations for all 20 topics were recorded along with p-values which were corrected for multiple comparisons at *p*<.05 using the Benjamini-Hochberg False Discovery Rate procedure.^37^

## Results

**Figure 1:**
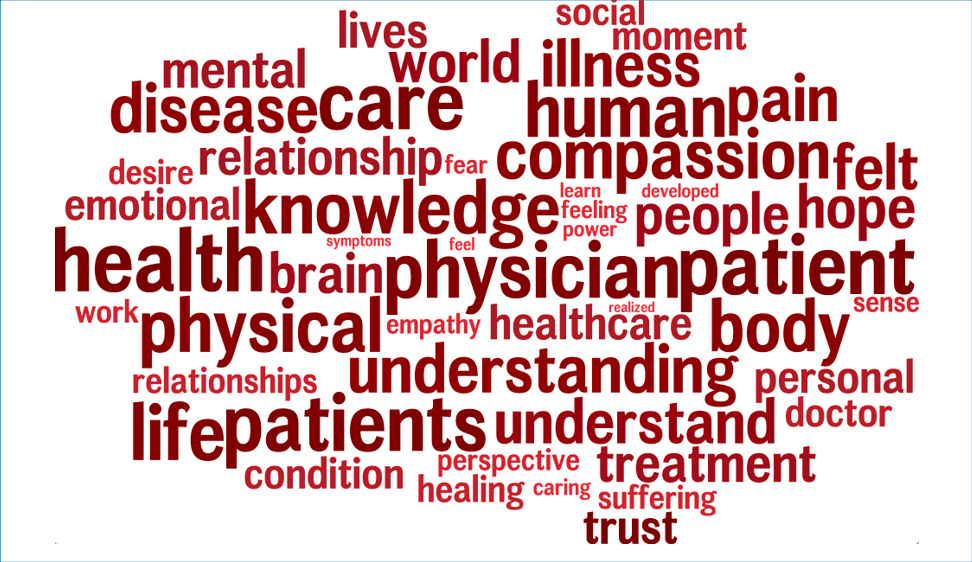
Language topic correlated with high physician empathy.

A linguistic topic was correlated with physician empathy (*r *= .074, *p* < .05), after correcting for multiple comparisons and controlling for gender. This topic consisted of words associated with key features of empathic engagement in patient care, such as "empathy", "understanding," "compassion," "perspective," "caring," and "trust." Figure 1 shows the language topic extracted by linguistic analysis of admission essays that were significantly correlated with scores on the JSE.

## Discussion

This study shows that some language used in medical school application essays predicts physician empathy. This finding could inform medical school admissions contexts, which are increasingly interested in selecting for more empathetic future physicians. Further, the observed linguistic findings provide insight into how empathy is expressed in language by future physicians more generally.

The words that were associated with empathy may suggest a primary focus on the experience of the patient. The top three words associated with empathy in our sample were "health," "patient," and "care." While these findings may seem nonspecific in a sample of students pursuing a career in medicine, they suggest an interest in patients rather than other aspects of medical practice such as technology, financial gain, professional prestige, or career-related motivations. This finding is interesting in the context of healthcare's current emphasis on patient-centered medicine. Where the physician's role was once to dictate a diagnosis and course of treatment, practitioners are now encouraged to understand and address the individual values and needs of patients in clinical contexts.^38^ Further research might explore how teaching patient-centered approaches to medicine impact the empathy of medical students in their clinical training. The other words associated with high empathy scores also reflect key components of empathic engagement in patient care such as "understanding," "compassion," "human," "feeling," "knowledge," and "trust." In general, our findings provide further support for current characterizations of empathy in healthcare as a cognitive attribute that involves understanding of patient's experiences, coupled with compassionate concern to minimize suffering.

These language results are in line with several specific findings in the research literature on physician empathy. Empathy in medical students is correlated with sociability,^38^ emotional intelligence,^39^ and conscientiousness.^40^ Empathic concern has been linked to prosocial behaviors such as higher rates of organ donation.^41^ More empathic healthcare practitioners also have more positive attitudes toward integrative care, and cooperative attitudes towards one another.^42^ Medical students nominated by their classmates for excellence in clinical competence had higher than average empathy scores.^43^ More empathic medical students also tended to choose people-oriented over technology-oriented specialties.^44^ These findings complement the language results in the present study by suggesting a link between higher levels of empathy and an orientation towards others, compassionate concern, and emotional intelligence.

Our study had several limitations. First, we had a relatively small sample size by linguistic analysis standards. While the study includes 1,805 participants, a larger than average sample size in most educational studies, many studies using computational linguistic analysis involve an order of magnitude more participants (closer to *N* = 10,000). Second, this study was conducted at a single private medical school in the northeast of the United States, so care should be taken when generalizing beyond this context, especially in regard to the international medical education community.

Figure 1. Language topic correlated with high physician empathy. This linguistic topic positively correlated with scores on the Jefferson Empathy Scale (*r* = .074, *p* < .05). Note that the larger size of the words indicates a higher correlation strength.

Third, while effect sizes are within standard ranges in linguistic analysis studies, they are relatively low in absolute magnitude. These small effect sizes may be due to a self-presentation bias in responses to the empathy scale and within the essays themselves, resulting in a ceiling effect which together may have constrained variance and decreased signal in the language data. In other words, the task of an admissions essay is to represent oneself in the best possible light; therefore, other more spontaneously generated sources of natural language might provide more variance in empathy and should be explored in future studies. Fourth, because our sample included students that were accepted and chose to attend medical school at a single institution, the variation could be limited by admission selection criteria.

Despite these limitations, the present study represents a step toward better understanding and selecting for more empathic medical students. Previous research has shown that there are no formal criteria for readers of medical school personal statements.^45^ In one study, ratings of personal statements had no predictive validity for future success.^46^ For these reasons, some have suggested that a more quantitative assessment should complement the essay reading process.^47^ While most self-report measures are subject to social desirability bias, linguistic analysis offers one method of bypassing some of these demand characteristics, particularly if applicants are not aware of the traits being considered or the language models that are associated with them.

Computational linguistic analysis is a method currently used in evaluating job applications at large companies^48^ and may soon be used for admissions purposes in many academic contexts. Computational linguistic models are capable of predicting scores related to a variety of outcome measures based on language alone.^49^ In other words, our findings suggest that an empathy score could, through future linguistic modeling and validation work, be automatically generated for each medical school admissions essay using this technology.

## Conclusions

Empathy assessments have received increasingly widespread attention in medical education. The language findings in the present study shed light on the words correlated with empathy and suggest that physician empathy can be identified in medical school admission essays using these methods. Demonstration of this technological capability to associate empathic orientation with linguistic features is the first step towards admissions committees selecting for more empathetic medical school applicants. Specific language themes identified in this study should be followed by future research to further specify their relationship with empathy. These linguistic insights may impact not only our understanding of physician empathy but inform selection committees responsible for medical student admissions.

### Acknowledgments

Contributors: The authors would like to thank Dr. Elizabeth Y. Brooks, DPM for her enthusiasm and support of this project as well as Dr. Martin E. P. Seligman, PhD.

Funders: This publication was made possible through the support of a grant from the Templeton Religion Trust. The opinions expressed in this publication are those of the authors and do not necessarily reflect the views of the Templeton Religion Trust. We also thank the Noguchi Medical Research Institute in Tokyo, Japan.

### Conflict of Interest

The authors declare that they have no conflict of interest.
